# Calculus of Appendix

**Published:** 1913-03

**Authors:** 


Calculus of Appendix. Dauriac and Desternes, *Bull. et Mem. de la Soc. de Med. de Paris,* October 27, 1912. The calculus removed from a youth of 19, was irregularly mulberry-shaped, yellowish, 2x1 c. m. It consisted of a homogeneous white center of carbonate and phosphate of calcium, surrounded by strata of yellowish mixtures of these salts with stercobilin. Letulle, in discussing, said that in an experience of about 600 microscopic examinations of appendices removed by operation and of about 2000 autopsies, he had found appendiceal calculus extremely rare.

